# Multifunctional targeted liposomal drug delivery for efficient glioblastoma treatment

**DOI:** 10.18632/oncotarget.17976

**Published:** 2017-05-18

**Authors:** Zakia Belhadj, Changyou Zhan, Man Ying, Xiaoli Wei, Cao Xie, Zhiqiang Yan, Weiyue Lu

**Affiliations:** ^1^ Department of Pharmaceutics, School of Pharmacy, Fudan University & Key Laboratory of Smart Drug Delivery (Fudan University), Ministry of Education, Shanghai 201203, P.R. China; ^2^ Department of Pharmacology, School of Basic Medical Sciences, Fudan University, Shanghai 200032, P.R. China; ^3^ State Key Laboratory of Medical Neurobiology & The Collaborative Innovation Center for Brain Science, Fudan University, Shanghai 200032, P.R. China; ^4^ Institute of Biomedical Engineering and Technology, Shanghai Engineering Research Center of Molecular Therapeutics and New Drug Development, School of Chemistry and Molecular Engineering, East China Normal University, Shanghai 200062, P.R. China; ^5^ State Key Laboratory of Molecular Engineering of Polymers, Fudan University, Shanghai 200433, P.R. China

**Keywords:** multifunctional liposomes, blood–brain barrier, blood–brain tumor barrier, glioma, pharmacodynamics

## Abstract

Glioblastoma multiforme (GBM) has been considered to be the most malignant brain tumors. Due to the existence of various barriers including the blood–brain barrier (BBB) and blood–brain tumor barrier (BBTB) greatly hinder the accumulation and deep penetration of chemotherapeutics, the treatment of glioma remains to be the most challenging task in clinic. In order to circumvent these hurdles, we developed a multifunctional liposomal glioma-targeted drug delivery system (c(RGDyK)/pHA-LS) modified with cyclic RGD (c(RGDyK)) and p-hydroxybenzoic acid (pHA) in which c(RGDyK) could target integrin α_v_β_3_ overexpressed on the BBTB and glioma cells and pHA could target dopamine receptors on the BBB. *In vitro*, c(RGDyK)/pHA-LS could target glioblastoma cells (U87), brain capillary endothelial cells (bEnd.3) and umbilical vein endothelial cells (HUVECs) through a comprehensive pathway. Besides, c(RGDyK)/pHA-LS could also increase the cytotoxicity of doxorubicin encapsulated in liposomes on glioblastoma cells, and was able to penetrate inside the glioma spheroids after traversing the *in vitro* BBB and BBTB. *In vivo*, we demonstrated the targeting ability of c(RGDyK)/pHA-LS to intracranial glioma. As expected, c(RGDyK)/pHA-LS/DOX showed a median survival time of 35 days, which was 2.31-, 1.76- and 1.5-fold higher than that of LS/DOX, c(RGDyK)-LS/DOX, and pHA-LS/DOX, respectively. The findings here suggested that the multifunctional glioma-targeted drug delivery system modified with both c(RGDyK) and pHA displayed strong antiglioma efficiency *in vitro* and *in vivo*, representing a promising platform for glioma therapy.

## INTRODUCTION

Although chemotherapy is an indispensable auxiliary treatment for malignant glioma, the clinical outcome is usually limited due to the specific properties of glioma, such as the highly infiltrative nature. In addition to the low efficacy of current drugs, drug delivery from the circulation to the brain is rigorously hampered by the blood brain barrier (BBB), and blood–brain tumor barrier (BBTB). Thus, researchers utilized various methods to conquer these barriers and achieved efficient glioma treatment [1–3]. Despite extensive efforts, the therapeutic efficiency of nanoparticulate drug delivery systems (NDDS) against glioma was still severely impaired due to two intrinsic limitations of nanodrugs, one is their limited blood circulation mainly due to recognition by the reticule endothelial system (RES) [4], which could cause sublethal tumor distribution of the anticancer drugs. The other is the poor tumor penetration of the conventional NDDS. The tumor penetration of nanoparticle (NP) is hurdled by the existence of several biological and pathological barriers including the dense extracellular matrix (ECM) and the elevated interstitial fluid pressure [5, 6].

A number of promising strategies have been developed for improving the delivery of chemotherapeutics to the brain and targeting to glioma such as receptor-, transporter-, or adsorption-mediated drug delivery according to different transport mechanisms [7, 8]. Therefore, developing brain-targeted drug delivery systems would be of great significance to improve the therapeutic effects and to reduce the side effects. Indeed, achieving brain tumor targeting drug delivery with reduced unwanted drug exposure to healthy organs has become the Holy Grail earnestly pursued in the medical community.

Glioblastoma comprises 80% of malignant brain tumors, which is a life-threatening risk due to rapid development or recurrence and poor prognosis [9, 10]. To achieve effective delivery to brain cancer, the drug delivery system has to cross the BBB first. Hence, a specific brain tumor targeting strategy must be set up. Benzamide analogues have high affinity with D1 and D2 dopamine receptors that are prominent in most parts of central nervous system [11]. Our group chose to use the small molecule ligand (p-Hydroxybenzoic Acid, pHA), through which pHA could bind to dopamine receptors overexpressed on the BBB. With the progression of brain tumor, angiogenesis and gradual impairment of the BBB, the BBTB emerges as the main obstacle to the transport of nanocarriers. The blood–brain tumor barrier (BBTB), similar to blood–brain barrier (BBB), is located between brain tumor tissues and microvessels formed by highly specialized endothelial cells (ECs), limiting the paracellular delivery of most hydrophilic molecules to tumor tissue [12]. Therefore, we also modified the drug delivery system with c(RGDyK), a well-known cyclic peptide that could bind preferentially to integrin α_v_β_3_ overexpressed on the BBTB and glioma cells [13].

Doxorubicin (DOX) is widely used as a chemotherapeutic agent against various solid tumors [14–16]. DOX can inhibit topoisomerase II (Topo II), an enzyme that can relax the DNA supercoils for transcription, by intercalating into DNA double strands via its planar aromatic ring, resulting in transcription repression [17].

**Figure 1:**
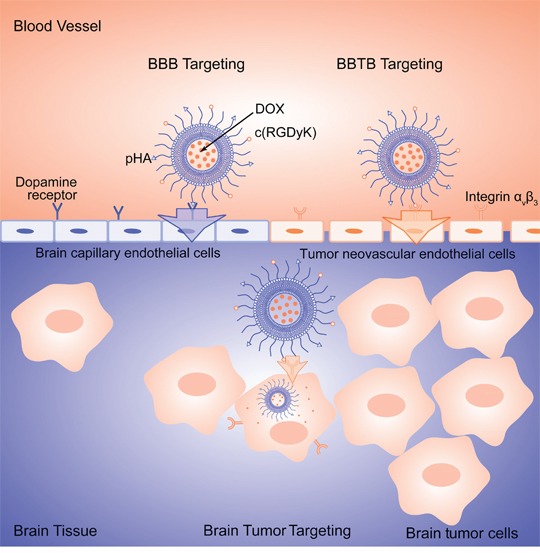
Schematic illustration of multifunctional DOX loaded c(RGDyK)/pHA-LS

In this present work, pHA and c(RGDyK) were both modified on the surface of PEGylated liposomes to develop multi-functional glioma-targeted drug delivery, while doxorubicin (DOX) was chosen as the chemotherapeutic agent for glioma therapy (Figure 1).

To elucidate the targeting ability, *in vitro* cellular uptake was performed on glioblastoma cells (U87), brain capillary endothelial cells (bEnd.3) and umbilical vein endothelial cells (HUVECs). The mechanism of cellular uptake was further elucidated using different inhibitors. *In vitro* BBB/BBTB crossing and tumor targeting ability of c(RGDyK)/pHA-LS were unambiguously performed. The *in vivo* glioma targeting and antiglioma efficacy of c(RGDyK)/pHA-LS were evaluated in intracranial glioma-bearing nude mice model.

## RESULTS

### Characterization of ligands modified PEG-DSPE

The thiolated ligands c(RGDyK)-SH, pHA-SH were synthesized by reaction of the amino-functionalized ligands (c(RGDyK)-NH_2_, pHA-NH_2_) with SATP. Since the SATP-introduced thiol is protected with an acetate group, the availability of thiol group was addressed by deprotection with hydrazine hydrate (N_2_H_4_.H_2_O). HPLC analysis and ESI-MS confirmed the purity and molecular weight (Supplementary Figure 1). The functional materials c(RGDyK)-PEG-DSPE and pHA-PEG-DSPE were obtained via the Michael-type addition reaction between thiol and maleimide groups. After the removal of excessive thiolated ligands by dialysis, the functionalized PEG-DSPE was lyophilized and subjected to ^1^H-NMR spectrometry. The characteristic peak of maleimide group at 6.7ppm disappeared in the ^1^H-NMR spectra of c(RGDyK)-PEG-DSPE and pHA-PEG-DSPE confirming the complete conversion of thiol group via Michael addition reaction (Supplementary Figure 2).

### Characterization of liposomes

The liposomes loaded with DOX with similar average size and narrow size distributions were successfully prepared. The encapsulation efficiency of DOX in LS/DOX, pHA-LS/DOX, c(RGDyK)-LS/DOX and c(RGDyK)/pHA-LS/DOX were 96.4±2.00, 95.48±2.16, 94.10±2.78, 95.47±1.52%. No obvious differences in encapsulation efficiencies and vesicle sizes were found among modified and unmodified liposomes, thus the ligand modified PEG-DSPE did not affect the physical properties of liposomes. The results of TEM image revealed that the liposomes were homogeneously spheroids (Supplementary Figure 3).

### Cellular selectivity of liposomes

**Figure 2:**
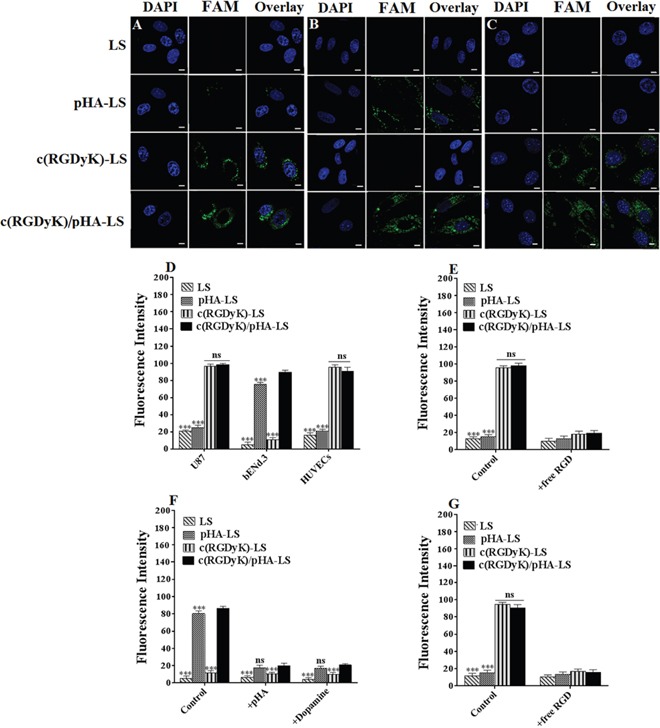
Cellular selectivity of liposomes Confocal images of cellular uptake of FAM-loaded LS at 37°C for 4h in U87 **(A)**, bEnd.3 **(B)** and HUVECs **(C)** (Scale bar=10μm). **(D)** Quantitative cellular uptake of different liposomes at 37°C for 4h in three kinds of cells measured by a flow cytometer. Competition assay for *in vitro* binding of liposomal formulations in U87 **(E)**, bEnd.3 **(F)** and HUVECs **(G)** at 4°C after incubation for 12h. Mean±SD, n=3, ***p<0.001, c(RGDyK)/pHA-LS versus other liposomal formulations.

The cellular uptake of c(RGDyK)/pHA-LS was investigated in glioma cells (U87), brain capillary endothelial cells (bEnd.3) and umbilical vein endothelial cells (HUVECs). Due to the potent cell penetration and multifunctional targeting ability, c(RGDyK)/pHA-LS displayed the highest cellular uptake efficiency in the three kinds of cell lines. In U87 and HUVECs cells, the uptake of the liposomes modified with both targeting moieties was 5.12-, 7.36-fold higher that of plain liposomes, meanwhile, the presence of c(RGDyK) motif increased the uptake of the c(RGDyK)/pHA-LS by 4.36- and 3.56-fold compared with that of pHA modified liposomes, respectively. Figure 2 displayed that the uptake of multifunctional targeting liposomes in bEnd.3 cells was 28.43- and 10.06-fold higher than that of unmodified and c(RGDyK) modified liposomes. The qualitative observation of the confocal images showed the same results (Figure 2). According to the cellular uptake results, liposomes modified with both c(RGDyK) and pHA possessed the brain targeting ability of pHA, neovasculature targeting ability and glioma targeting ability of c(RGDyK).

The competitive inhibition of binding of c(RGDyK)/pHA-PEG-DSPE incorporated liposomes on U87, bEnd.3 and HUVECs cells at 4°C after treatment with different inhibitors was evaluated quantitatively by a flow cytometer. When U87 cells were pre-incubated with an excess of c(RGDyK) peptide (100μM), the cell-binding of c(RGDyK)-LS (96.50%), c(RGDyK)/pHA-LS (98.18%) down to (18.28, 19.55%), respectively. The pre-incubation of free RGD peptide also decreased the binding of c(RGDyK)-LS, c(RGDyK)/pHA-LS in HUVECs (reduced from 94.04, 90.52 to 17.17, 15.60%, respectively). In bEnd.3 cells, the cell-binding of multi-functional targeting liposomes c(RGDyK)/pHA-LS (86.27%) and those modified with pHA (80.30%) was significantly decreased after pre-incubation with excess pHA or dopamine at 4°C (down to 20.09, 17.64 and 20.85, 16.73%), respectively. These results indicated that c(RGDyK) on the surface of liposomes increased the cellular association of multifunctional targeting liposomes c(RGDyK)/pHA-LS by specifically binding to the integrin (α_v_β_3_ might be closely involved) expressed on glioma cells (U87), and umbilical vein endothelial cells (HUVECs). Besides, c(RGDyK)/pHA-LS was associated with brain capillary endothelial cells (bEnd.3) through pHA-dopamine special binding pathway.

### Transport efficiency across *in vitro* BBB and BBTB models

**Figure 3:**
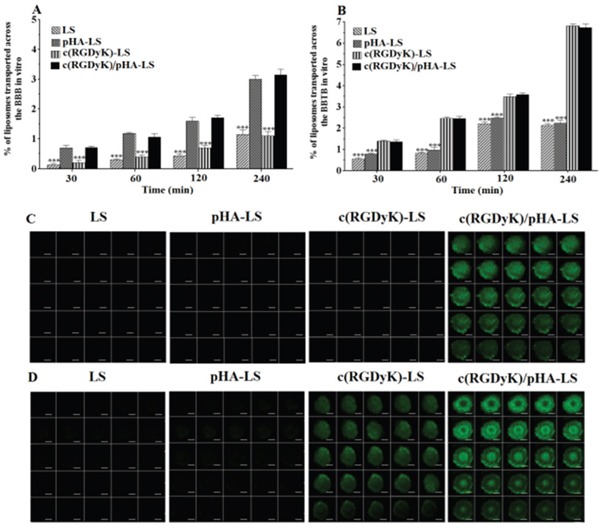
Multi-targeting ability *in vitro* Transcytosis efficiency of FAM-loaded LS, pHA-LS, c(RGDyK)-LS, and c(RGDyK)/pHA-LS *in vitro* BBB model **(A)** and BBTB model **(B)**. Targeting ability of different liposomes through *in vitro* BBB/U87 tumor spheroid and BBTB/U87 tumor spheroid co-culture model for 4h **(C, D)**. Mean±SD, n=3, ***p<0.001, c(RGDyK)/pHA-LS versus other liposomal formulations. (Scale bar=100μm).

The percentage of liposomes transported across the *in vitro* BBB model over a period of 4h was shown in Figure 3A. After 4h, 3.04±0.14% of pHA modified liposomes and 3.19±0.10% of the liposomes modified with both of c(RGDyK) and pHA transported across the BBB, which was evidently higher than that that of unmodified liposomes (1.18±0.14%) and those modified with c(RGDyK) (1.13±0.16%).

The BBTB model was also established to evaluate the transcytosis efficiency of the liposomes *in vitro*. As shown in Figure 3B, the presence of c(RGDyK) on the surface of liposomes significantly increased the transport of c(RGDyK)-LS and c(RGDyK)/pHA-LS than that of unmodified liposomes and pHA-LS as evidenced by their transport percentages (6.82±0.02, 6.74±0.12, 2.14±0.08, 2.23±0.15%), respectively.

### Multi-targeting ability *in vitro*

In the aspect of simulating the *in vivo* glioma microenvironment, the BBB/U87 tumor spheroids and BBTB/U87 tumor spheroids co-culture models were constructed and used to investigate the penetrating capabilities of various liposomes. As shown in Figure 3C, 3D, c(RGDyK)/pHA-LS displayed intense fluorescence and extensive penetration inside the spheroids after crossing the *in vitro* BBB or BBTB monolayers, and confocal microscopic measurement showed that c(RGDyK)/pHA-LS penetrated significantly deeper after traversing the BBB monolayers than did liposomes modified with a single ligand (either pHA or c(RGDyK)). Moreover, both c(RGDyK)/pHA-LS and c(RGDyK)-LS could cross the *in vitro* BBTB monolayer and internalize into the U87 tumor spheroids. These findings suggested that c(RGDyK)/pHA-LS possessed a noteworthy property in tumor cell targeting and intratumor penetration after crossing *in vitro* BBB or BBTB monolayers.

### Multi-targeting ability *in vivo*

**Figure 4:**
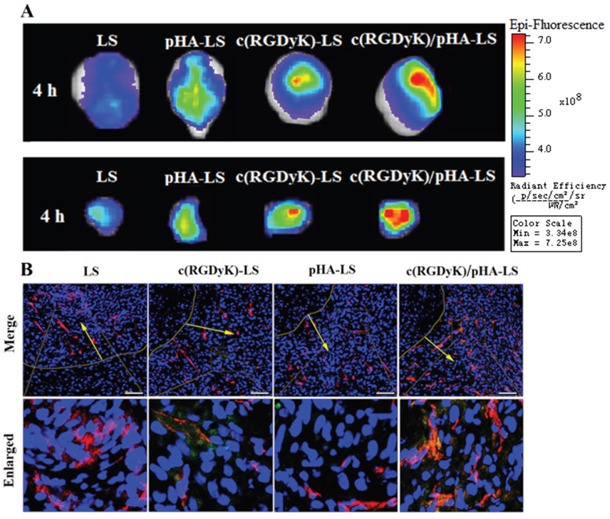
*In vivo* targeting ability of targeted liposomes *Ex vivo* imaging and semi-quantitative analysis of the fluorescence intensity of brains (top) and dissected tumors (bottom) 15 days after implantation **(A)**. **(B)** Brain distribution of FAM-loaded liposomes from nude mice bearing intracranial U87, 15 days post-implantation and 4h post injection. Nuclei were stained by DAPI (blue), and microvessels were stained by CD31 antibody (red), while green represents the liposomes. (Scale bar=100μm).

In order to study the *in vivo* brain targeting efficiency, nude mice bearing intracranial glioma were treated with different DiR-labeled liposomes, and then we performed *ex vivo* imaging of brains, tumor tissues and the main organs 4h after the injection of different DiR-labeled liposomes. Compared with plain liposomes (LS/DiR), c(RGDyK)-LS/DiR and pHA-LS/DiR groups, the fluorescence signal in the tumor-bearing brain of c(RGDyK)/pHA-LS group was much stronger at 4h post-injection. As shown in Figure 4A, top, unmodified liposomes displayed weak fluorescence signal, while pHA-LS distributed widely in the whole brain owing to its BBB targeting efficiency. In contrary, c(RGDyK)-LS exhibited slight distribution in the brain tumor. Meanwhile, the liposomes modified with both c(RGDyK) and pHA also had wide distribution in the glioma tissue (Figure 4A, bottom). This proved that c(RGDyK)/pHA-LS successfully overcame the *in vivo* BBB and BBTB delivery barriers, and accumulated in the glioma.

The most important observation of distribution in other tissues was the high fluorescence intensity in liver of mice received c(RGDyK)-LS and c(RGDyK)/pHA-LS (Supplementary Figure 4), which should be attributed to the increased phagocytosis of liposomes through mononuclear phagocyte system caused by c(RGDyK) on the surface of liposomes [18].

We further conducted *in vivo* brain distribution analysis to confirm the precise glioblastomas targeting property of liposomes. As expected, c(RGDyK)/pHA-LS exhibited higher accumulation and deeper distribution in the brain tumor area (Figure 4B). Nearly no fluorescence of LS and pHA-LS was observed in the tumor. c(RGDyK)-LS showed a weak fuorescence in glioma due to the poor targeting effect. Notably, *in vivo* distribution of c(RGDyK)/pHA-LS was found predominantly in the tumor region, which was in consistence with the results of *ex vivo* imaging, indicating better glioma targeting efficiency.

### *In vitro* cytotoxicity

**Figure 5:**
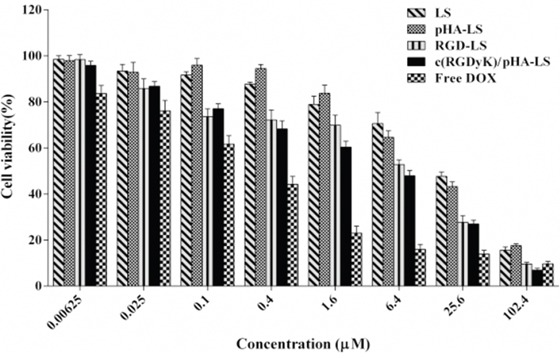
Antiproliferative effect of free DOX and various DOX-LS on U87 glioma cell The cell viability was examined by MTT assay and expressed as a percentage (mean±SD, n=3).

DOX-loaded liposomes were tested in U87 cells for evaluation of their *in vitro* antiproliferation effect (Figure 5). The U87 cell viability was inhibited in a concentration dependent manner. The IC_50_ value of free DOX was 0.24μM, of c(RGDyK)/pHA-LS/DOX was 4.79μM, of c(RGDyK)-LS/DOX was 6.92μM, of pHA-LS/DOX and LS/DOX was respective 16.22, and 19.50μM. In comparison to DOX liposomes, the IC_50_ value of free DOX was the lowest due to quick cellular uptake and accumulation of small molecule chemotherapeutics during 4h treatments [19]. Among all of the different DOX-loaded liposomes, c(RGDyK)/pHA-LS induced the strongest antiproliferation effect on U87 cells, indicating that the *in vitro* growth inhibitory effect of liposomal DOX was enhanced by modifying the liposome surface with c(RGDyK) and pHA.

### *In vivo* antitumor efficacy in glioma-bearing mice

**Figure 6:**
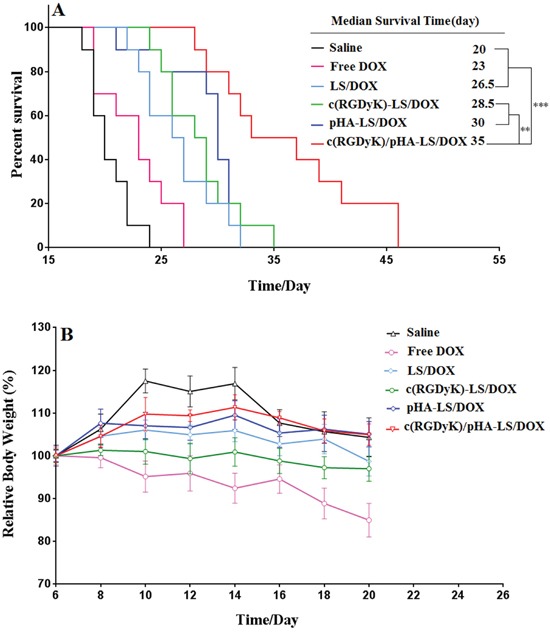
*In vivo* therapeutic efficacy of DOX-loaded liposomes **(A)** Kaplan–Meier survival curve of mice bearing intracranial U87 glioma, treated with different DOX formulations at day 7, 9, 11, 13, and 15 post-implantation. The median survival time of mice treated with c(RGDyK)/pHA-LS was significantly longer than that of mice treated with physiological saline (p<0.001), free DOX (p<0.001), LS-DOX (p<0.001), c(RGDyK)-LS/DOX (p<0.01) and pHA-LS-DOX (p<0.01). **(B)** Change of mice body weight (n=10).

The therapeutic effect of the various DOX loaded liposomal formulations was investigated on the orthotropic glioma model. As showed in Figure 6, the median survival time of saline group, free DOX group, LS/DOX, c(RGDyK)-LS/DOX, pHA-LS/DOX, and c(RGDyK)/pHA-LS/DOX were 20, 23, 26.5, 28.5, 30, and 35 days, respectively. The group given c(RGDyK)/pHA-LS displayed the longest survival time among all of the groups, with very statistical significance (1.50-, 1.76- and 2.31-fold) higher than that of pHA-LS, c(RGDyK)-LS and LS, respectively. This demonstrated the great potential of the dually modified liposomes under the multi-targeting mechanism.

### Safety evaluation of DOX-loaded liposomes

Cardiotoxicity is one of the main side effects for anthracycline as reported, and daily injection of DOX can cause weight loss and cardiotoxicity [20, 21]. Thus the systemic toxicity of drug-loaded liposomes was evaluated here in healthy mice. As shown in Supplementary Figure 5, intravenous administration of free DOX caused noticeable cardiac tissue degeneration, necrosis and slightly edema. The HE staining also demonstrated that no evidence of abnormal and inflammatory cell infiltration in the heart sections and in the other main organs were observed among all of the liposomes treated groups (Supplementary Figure 5). Besides, no significant change was noted of body weight in all DOX-loaded liposome treatment groups. While free DOX caused more weight reductions presumably due to the cardiotoxicity (Figure 6). These results clearly demonstrated the safety and biocompatibility of a liposomal drug delivery system at the treatment dose.

## DISCUSSION

Glioblastoma, accounting for 29% of all primary central nervous system tumors, severely threaten human health for fast development and poor prognosis [10]. Improvement in chemotherapy against glioblastoma has not been translated into a meaningful improvement in patient outcome due to the limited drug permeability across the BBB/BBTB, and the low accumulation of the drug into the tumor parenchyma [22, 23]. Thus, the growing awareness of this fact underscores the importance of pursuing more rational drug delivery strategies.

Single-targeting systems often show inadequate efficiency and specificity to brain tumor [24]. Gao et al. functionalized polymeric nanoparticles with RGD and IL-13p for the dual targeting of neovasculature and GBM cells, dual modification enhanced uptake by endothelial cells and brain tumor cells [25, 26]. However, these researches mainly focused on targeting the brain tumor neovessels and brain tumor cells. Nevertheless, they lacked the BBB targeting. Hence, we developed a multifunctional drug delivery system which could circumvent three barriers (BBB, BBTB and glioma cells) with the purpose of enhancing the antiglioma efficacy *in vivo*.

In this context, multiple targeting strategies have been recently explored into the glioma therapy with the purposes of circumventing the BBB/BBTB and achieving glioma targeting [27–30]. Multifunctional liposomes could be a boost for brain delivery efficiency. The multi-targeting mechanisms can be achieved by incorporation of two or more kinds of ligands that can traverse the BBB, BBTB and target glioma cells. In this work, we developed multifunctional targeting liposomes modified with two targeting moieties in which the small molecule pHA could bind to dopamine receptors expressed on the BBB, and the stable cyclic arginine-glycine-aspartic acid peptide-c(RGDyK) on the surface of liposomes could enhance the BBTB transcytosis and tumor cells targeting [31–33]. Liposomes were chosen as the drug carrier since they are nontoxic, nonimmunogenic and, coating the surface of liposomes with PEG provides ‘stealth’ properties and greatly prolongs circulation [34].

Because this study was mainly focused on the development of systemic glioma-targeted drug delivery systems capable to achieve efficient BBB/BBTB penetrating and glioma cells targeting, the targeting ability of c(RGDyK)/pHA-LS was investigated on the BBB, BBTB models *in vitro* and *in vivo*. In this study, bEnd.3 cells, U87 cells and HUVECs were selected as the models of BBB, glioma and BBTB *in vitro*, respectively. The cellular uptake of liposomes by three kinds of cells was estimated through confocal laser microscopy and flow cytometry *in vitro*. Compared to liposomes modified with a single peptide ligand, the liposomes modified with both c(RGDyK) and pHA displayed significant uptake in glioma cells, brain capillary endothelial cells and umbilical vein endothelial cells.

The assay of transport across the BBB and BBTB indicated that c(RGDyK)/pHA modification on the drug-loaded liposomes plays a major role in the transport of the multi-targeting liposomes across the BBB and BBTB models. pHA modification enhanced the transport of liposomes across the BBB as proved by the results of inhibition assay, while the existence of c(RGDyK) on the surface of liposomes enhanced the transcytosis efficiency of the multifunctional targeting liposomes across the BBTB.

To evaluate the multi-targeting effect *in vitro*, the BBB/U87 tumor spheroids and BBTB/U87 tumor spheroids co-culture model were established for simulating the situation *in vivo*. The results strongly confirms those obtained from the transport across the BBB, BBTB *in vitro* and showed higher penetrability of the multifunctional targeting liposomes. The advantages of RGDyK) ligand modification on the surface of liposomes include its high binding affinity to integrin α_v_β_3_ expressed on both BBTB and glioma cells, this property enhances the tumor cell targeting and intratumor penetration of multifunctional targeting liposomes. Therefore, c(RGDyK)/pHA-LS was likely to achieve the significant delivery efficiency compared to that of liposomes modified with a single ligand.

To further understand the benefits of this novel drug delivery strategy, *in vivo* fluorescence imaging study was conducted. Distribution of c(RGDyK)/pHA-LS in the glioma site was much more than other groups, which denoted that c(RGDyK)/pHA-LS had a strong glioma targeting effect. It was suggested that c(RGDyK)/pHA-LS exhibited efficient multi-targeting effect: small molecule ligand pHA mediated the transport of dually modified liposomes across the BBB through dopamine receptors, (RGDyK) modification boosted the transcytosis of targeted liposomes across the BBTB by specifically binding to the integrin (possibly α_v_β_3_) overexpressed on the tumor neovasculature as well as glioma cells.

*In vitro* cytotoxicity of DOX to U87 glioma cells was effectively enhanced following the modification of DOX-loaded liposomes with both c(RGDyK) and pHA. The results from MTT assay (Figure 5) indicated that c(RGDyK)/pHA-LS evidently decreased the IC_50_ value. The improved anti-proliferation effect of multifunctional targeting liposomes was believed to be attributed to the existence of c(RGDyK) peptide, which facilitated the internalization of the formulation into the U87 glioma cells.

In the survival study, the group of mice administered c(RGDyK)/pHA-LS lived significantly longer than the other groups. Such enhanced antiglioma efficacy of c(RGDyK)/pHA-LS could be probably owing to the c(RGDyK)/pHA functionalization, which could facilitate the accumulation of more liposomes in the tumor site, and further drive DOX-loaded liposomes internalization into glioma cells. Thus, the systemic safety of DOX-loaded liposomes modified with c(RGDyK) and pHA was also good.

Therefore, the current investigation could provide insight on the rational design of brain-targeting systems. Moreover, it is worthwhile to explore multifunctional targeting drug delivery system for further enhancing the efficiency of brain delivery.

## MATERIALS AND METHODS

### Materials

Fmoc-protected amino acids were purchased from GL Biochem Ltd (Shanghai, China). HSPC (hydrogenated soy phosphatidylcholine) and mPEG_2000_-DSPE were obtained from Lipoid GmbH (Ludwigshafen, Germany). Cholesterol was supplied by Sinopharm Chemical Reagent Co. Ltd. (Shanghai, China). Near infrared dye DiR was obtained from Invitrogen, USA. Sephadex G50 and 5-carboxyfluorescein (FAM) were provided by Sigma (St. Louis, MO). Doxorubicin hydrochloride was supplied by Dalian Meilun Biotech Co., Ltd (Dalian, China). Rat tail collagen Type I was purchased from Shengyou Biological Technology Co. (Hangzhou, China). DNase I and collagenase were supplied by Dingguo Biological Technology Co. Ltd (Shanghai, China).

Human glioblastoma cells (U87), human umbilical vascular endothelial cells (HUVECs) and brain capillary endothelial cells (bEnd.3) were obtained from Shanghai Institute of Cell Biology, cultured in special Dulbecco’s modified Eagle medium (Gibco) supplemented with 10% fetal bovine serum (FBS, Gibco). ICR mice and BALB/c nude mice aged 4-6 weeks were supplied by Shanghai SLAC Laboratory Animal Co. Ltd (Shanghai, China) and housed under SPF conditions. All animal experiments were carried out in accordance with the guidelines evaluated and approved by the Ethics Committee of Fudan University.

### Synthesis and characterization of c(RGDyK)-PEG-DSPE and pHA-PEG-DSPE

The thiolated ligands c(RGDyK)-SH, pHA-SH were synthesized by reaction of the amino-functionalized ligands (c(RGDyK)-NH_2_, pHA-NH_2_) with SATP (N-Succunimidyl-S-acetyl thiopropionate) followed by deprotection with hydrazinium hydrate (N_2_H_4_.H_2_O) as shown in Supplementary Figure 6. The obtained thiolated ligands c(RGDyK)-SH and pHA-SH were respectively coupled with maleimide group of Mal-PEG_3400_-DSPE. Briefly, Mal-PEG_3400_-DSPE in DMF and thiolated ligand in PBS (0.1 M, pH=7.2) were reacted at room temperature with a molar ratio of 1:1.5. The excessive thiolated ligand was removed by dialysis (MWCO 3.5kDa). The yielded products were then confirmed by ^1^H-NMR.

### Preparation and characterization of liposomes

Different liposomal formulations loaded with DOX, FAM or DiR were prepared by the thin-film hydration and extrusion method as reported previously [35]. The constituents including HSPC, cholesterol, mPEG-DSPE, or/and c(RGDyK)-PEG-DSPE, or/and pHA-PEG-DSPE, or/and c(RGDyK)-PEG-DSPE/pHA-PEG-DSPE at the molar ratio of 55:40:5 for PEGylated liposomes (LS), 55:40:3:2 for single-ligand modified liposomes (c(RGDyK)-LS, pHA-LS) and 55:40:1:2:2 for multifunctional targeting liposomes (c(RGDyK)/pHA-LS) in CHCl_3_ solution were rotary evaporated to form a thin film. The dried film was subsequently hydrated with FAM solution in 60°C water bath for 2h, and extruded through a series of polycarbonate membranes (Whatman PLC., UK) with the pore size ranging from 200nm down to 50nm on an Avanti Mini-Extruder (Avanti Polar Lipids Inc). The unencapsulated FAM was removed by gel filtration over a Sephadex G-50 column with normal saline. For preparation of DiR-loaded liposomes, DiR was included in the lipid mixture and the thin film was then hydrated in saline. We prepared different DOX-loaded liposomes using a conventional ammonium sulfate gradient loading method as reported previously [36]. The particle size distributions of various liposomes were determined by the dynamic light scattering method (Nicomp 380ZLS, USA). The morphology of liposomes was observed by transmission electron microscope (TEM) (H-7000, Hitachi, Japan).

### *In vitro* cellular uptake study

U87, bEnd.3 and HUVECs cells were seeded into 12-well plates and incubated under 37°C for 24h. Then the cells were incubated with 5μM FAM-loaded liposomes of different formulations in DMEM supplemented with 10% FBS for 4h. The cells were imaged by confocal microscope and quantitatively analyzed by a flow cytometer test (FACS Aria, BD, USA).

In order to study the mechanism of c(RGDyK)/pHA modified liposomes, U87 and HUVECs cells were preincubated with a 20-fold molar excess of c(RGDyK) peptide, meanwhile bEnd.3 cells were preincubated with a 20-fold molar excess of pHA or dopamine for 2h at 4°C. After 2h pre-incubation with the inhibitors above, LS/FAM, c(RGDyK)-LS/FAM, pHA-LS/FAM, c(RGDyK)/pHA-LS/FAM were added in culture medium with 10% FBS for 12h at the concentration of 5μM (FAM) at 4°C. The fluorescence labeled cells were counted by a flow cytometer (FACS Aria, BD, USA).

### Transport of liposomes across the BBB and BBTB models

The BBB model was constructed according to previous reports [37, 38]. The isolated rat primary brain capillary endothelial cells were seeded on the apical chamber of Transwell coated with rat tail collagen. Before starting the experiment, the transendothelial electrical resistance (TEER) was measured using an epithelial volt-Ωm (Millicel- RES, Millipore, USA) to monitor the tightness of the monolayer. Only cell monolayers with TEER above 200Ω· cm2 were used to evaluate the transport of liposomes across the BBB. FAM-loaded liposomes in DMEM with 10% FBS at the concentration of 30μM (FAM) were added into the apical chamber in advance. After 0.5, 1, 2, 4h, the samples taken from the lower compartment were determined by a fluorescence spectrophotometer (Cary Eclipse, Agilent, Australia).

To evaluate the transport of liposomes across the BBTB model, HUVECs/U87 co-culture model was constructed as previously reported [39]. Briefly, HUVECs were seeded in the apical chamber of Transwell and U87 cells were plated into the basolateral chamber at a 1:5 HUVECs/U87 ratio. Three days later, different FAM-loaded liposomes (LS, pHA-LS, c(RGDyK)-LS and c(RGDyK)/pHA-LS, at the final concentration of FAM 30μM) were added to the upper chamber of the transwells. At the time points of 0.5, 1, 2, and 4h incubation at 37°C, the fluorescence intensity of different formulations was detected by a fluorescence spectrophotometer.

### Multi-targeting ability *in vitro*

For further evaluating the multi-targeting ability of c(RGDyK)/pHA-LS, BBB/U87 tumor spheroids and BBTB/U87 tumor spheroids co-culture models were established. U87 tumor spheroids were established as follows: first, a 48-cell culture plate was coated with agarose-based DMEM (2% w/v), then 2×10^3^ U87 cells/400μL per well were plated on the pre-coated 48-well plate and incubated at 37°C for 10 days. Then U87 tumor spheroids were transferred into a 24-well plate and the Transwell basolateral chamber of BBB model established as previously were placed above the spheroids. For evaluating the targeting efficacy of the functionalized liposomes in BBTB/U87 tumor spheroids coculture model, the transwells of BBTB model were inserted above the tumor spheroids previously cultured for 10 days. In each apical chamber, FAM-loaded liposomes of LS, pHA-LS, c(RGDyK)-LS and c(RGDyK)/pHA-LS were added at a concentration of 30μM in DMEM with 10% FBS for 4h incubation, the tumor spheroids were washed with PBS and fixed with 4% paraformaldehyde for 30min, then subjected to confocal laser microscopy.

### Multi-targeting ability *in vivo*

The *in vivo* multi-targeting ability was evaluated through near-infrared *in vivo* imaging study of intracranial glioma-bearing mice. The U87 cells (5×10^5^cells) were implanted into the right striatum (1.8mm lateral, 0.6mm anterior to the bregma and 3mm of depth) of male BALB/c nude mice using a stereotactic fixation device with a mouse adaptor [40]. After inoculation, the mice were kept under the standard housing conditions for 15 days. The intracranial glioma bearing mice were then given 100μL DiR (0.25mg/kg) loaded liposomes via the tail vein. Four hours later, the *in vivo* fluorescence imaging was performed with an IVIS spectrum system (PerkinElmer, Waltham, MA).

The intracranial glioma bearing mice were also injected with 0.5mg/kg of FAM-loaded liposomes, 15 days after tumor implantation. After 4h, the mice were sacrificed, heart perfused with saline, and 4% paraformaldehyde sequentially. The brains were then harvested and further fixed in 4% paraformaldehyde. After 24h, each brain was frozen sectioned into 10μm thickness and subjected to confocal laser scanning microscopy after nuclei stained with DAPI and microvessels stained with anti-CD31 antibody.

### *In vitro* cytotoxicity assay

The *in vitro* activity of different DOX-LS against U87 glioma cells was determined by MTT assay as described previously [41]. The cells were seeded into 96-well plates at a density of 3×10^3^ cells per well. After attachement for 24h, various concentrations of DOX liposomes and free DOX were added to the wells for 72h of incubation. Thereafter, cells were exposed to 20μL of MTT (5mg/mL) in each well and incubated for 4h. The culture medium was removed and replaced with 150μL of dimethyl sulfoxide. Then, the absorbance was measured by a microplate reader (Power Wave XS, Bio-TEK, USA) at the wavelength of 490nm.

### *In vivo* antiglioma therapy

The mice bearing intracranial glioma were randomly divided into six groups (n=10 per group), receiving normal saline (NS), free DOX, LS/DOX, c(RGDyK)-LS/DOX, pHA-LS/DOX and c(RGDyK)/pHA-LS/DOX via the tail vein on the day 7, 9, 11, 13 and 15 after glioma inoculation with a single doxorubicin dose of 2mg/kg. Animals were monitored for body weight every 2 days. The survival times were recorded.

### *In vivo* safety evaluation

To investigate the safety evaluation of doxorubicin liposomes, tumor-free BALB/c mice (18–20g) were intravenously injected with saline, free DOX, LS/DOX, c(RGDyK)-LS/DOX, pHA-LS/DOX and c(RGDyK)/pHA-LS/DOX at the DOX dose of 2mg/kg for every other day for a total of five injections. A total of 24h after the last intravenous dosing of different formulations, the mice were sacrificed, and the major organs including heart, liver, spleen, lung, kidney, and brain were also collected for H&E histological analysis.

### Statistical analysis

Data are presented as the means± SD. The IC_50_ values were calculated by nonlinear regression analysis with the GraphPad Prism 6.0 version program. Two-way ANOVA analysis was used to determine the significance among groups. Survival data were presented using Kaplan-Meier plots and were analyzed using GraphPad Prism 6.0. For statistical analysis of all data, p<0.01 was considered as the lowest acceptable threshold for significance.

## SUPPLEMENTARY MATERIALS FIGURES


