# Supplementary material for: Multifunctional targeted liposomal drug delivery for efficient glioblastoma treatment
Source: Oncotarget. 2017 May 18;8(40):66889–900. doi: 10.18632/oncotarget.17976 (PMC5620143; doi:10.18632/oncotarget.17976)
Supplement: Supplementary file 1 [file oncotarget-08-66889-s001.pdf]

Multifunctional targeted liposomal drug delivery for efficient glioblastoma treatment

SUPPLEMENTARY MATERIALS

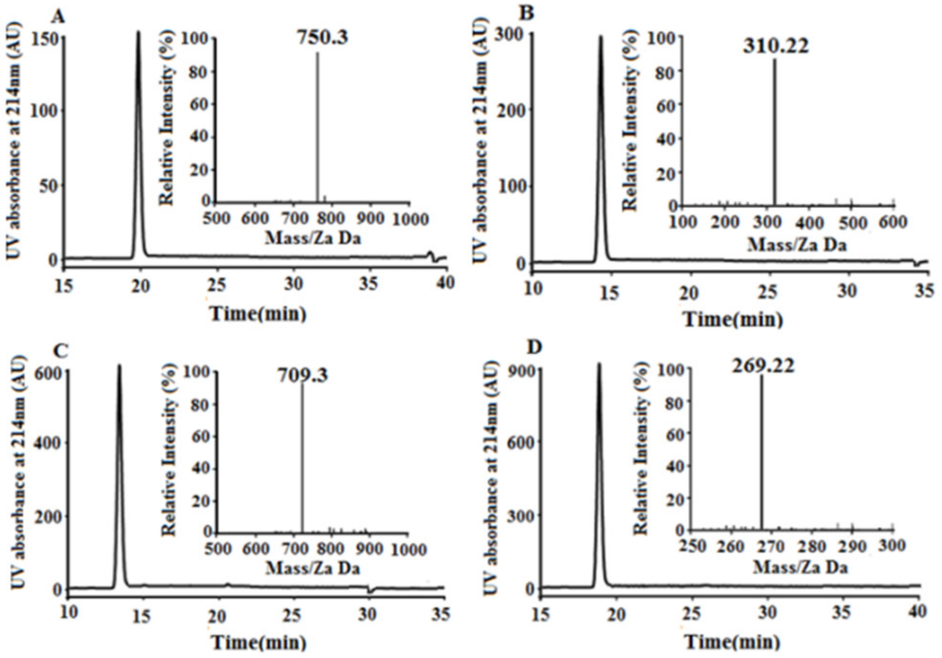

Supplementary Figure 1: Characterization of thiolated ligands. HPLC and ESI-MS spectrum of c(RGDyK)-SATP **(A)**, pHA-SATP **(B)**, c(RGDyK)-SH **(C)** and pHA-SH **(D)**.

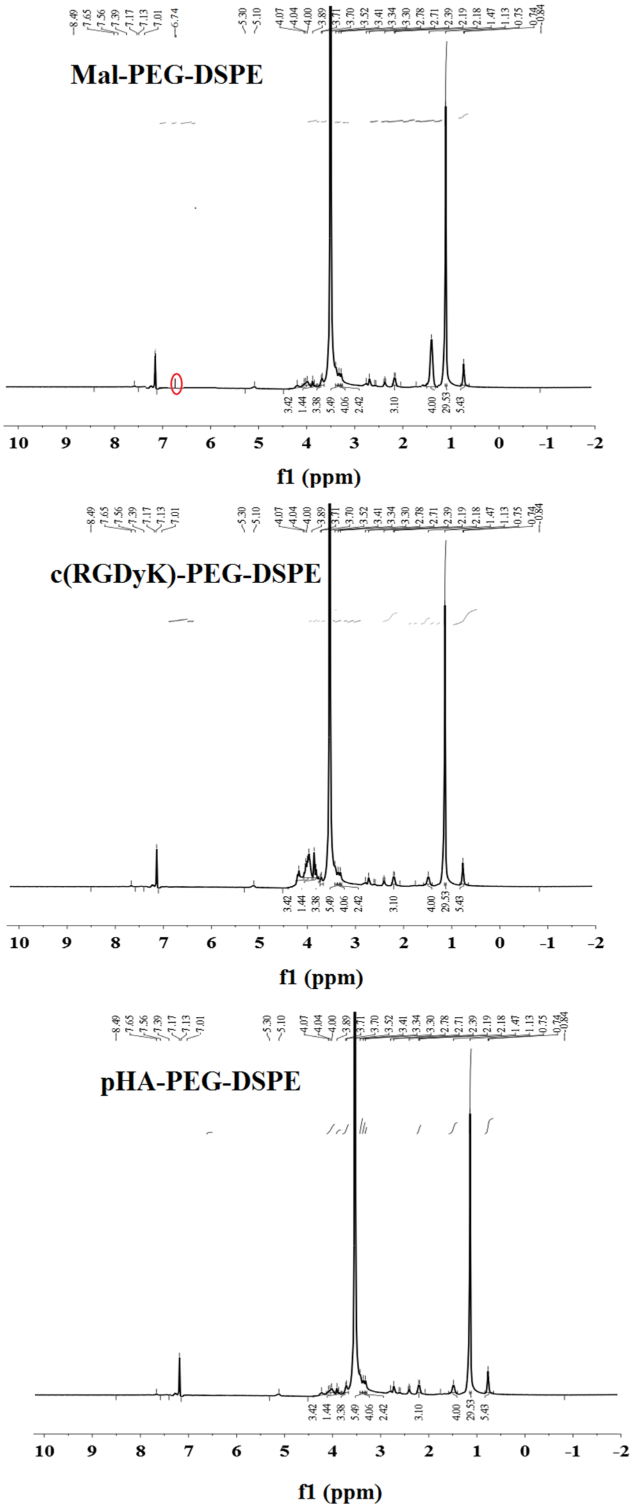

Supplementary Figure 2: Characterization of ligands modified PEG-DSPE. NMR spectra of Mal-PEG-DSPE, c(RGDyK)-PEG-DSPE and pHA-PEG-DSPE. The red circle highlights the characteristic peak of maleimide at 6.7 ppm in Mal-PEG-DSPE.

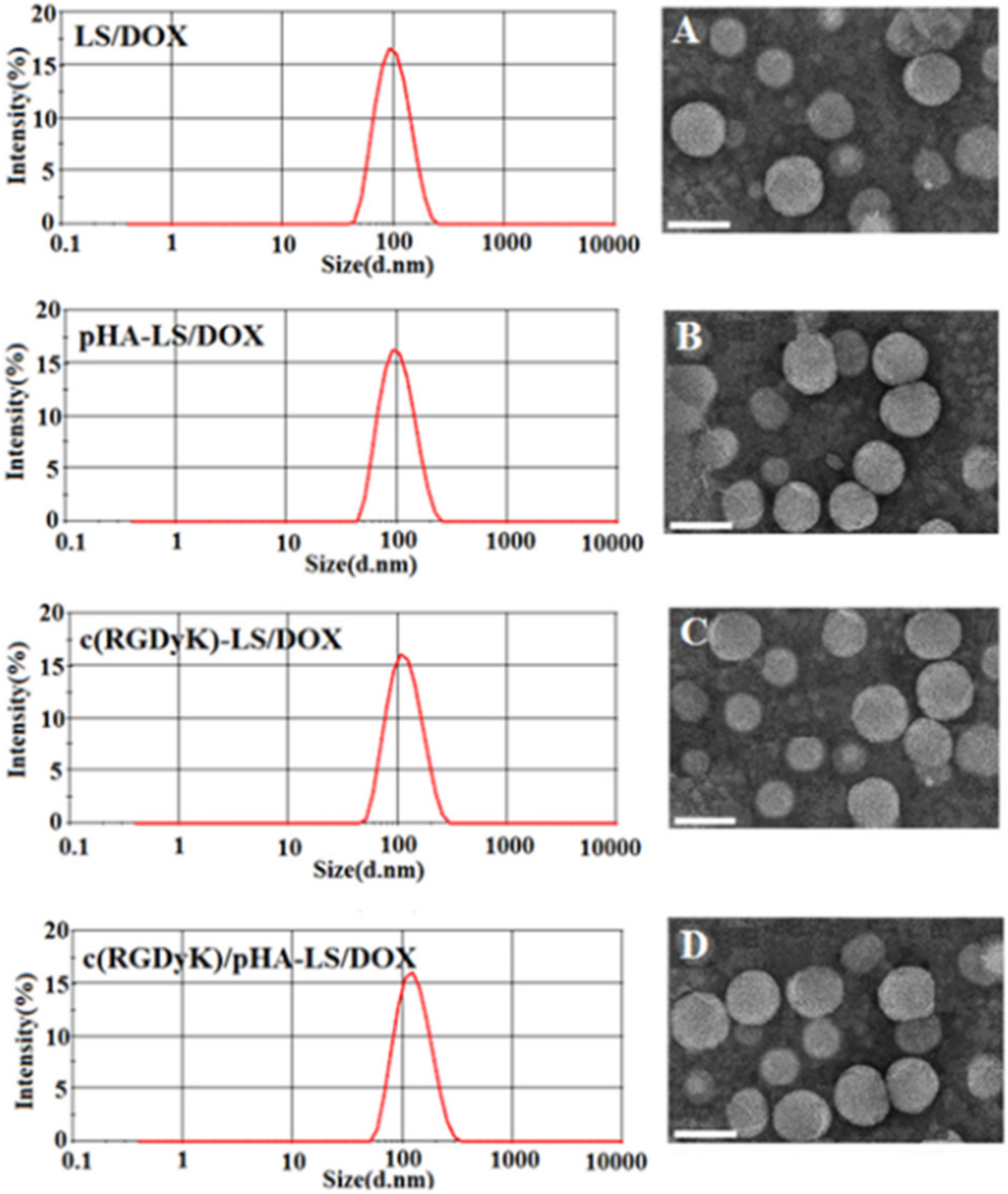

Supplementary Figure 3: Characterization of different DOX-loaded liposomes. Size distribution and Transmission Electron Microscopy images of LS/DOX **(A)**, pHA-LS/DOX **(B)**, c(RGDyK)-LS/DOX **(C)**, c(RGDyK)/pHA-LS/DOX **(D)**. (Scale bar=100nm).

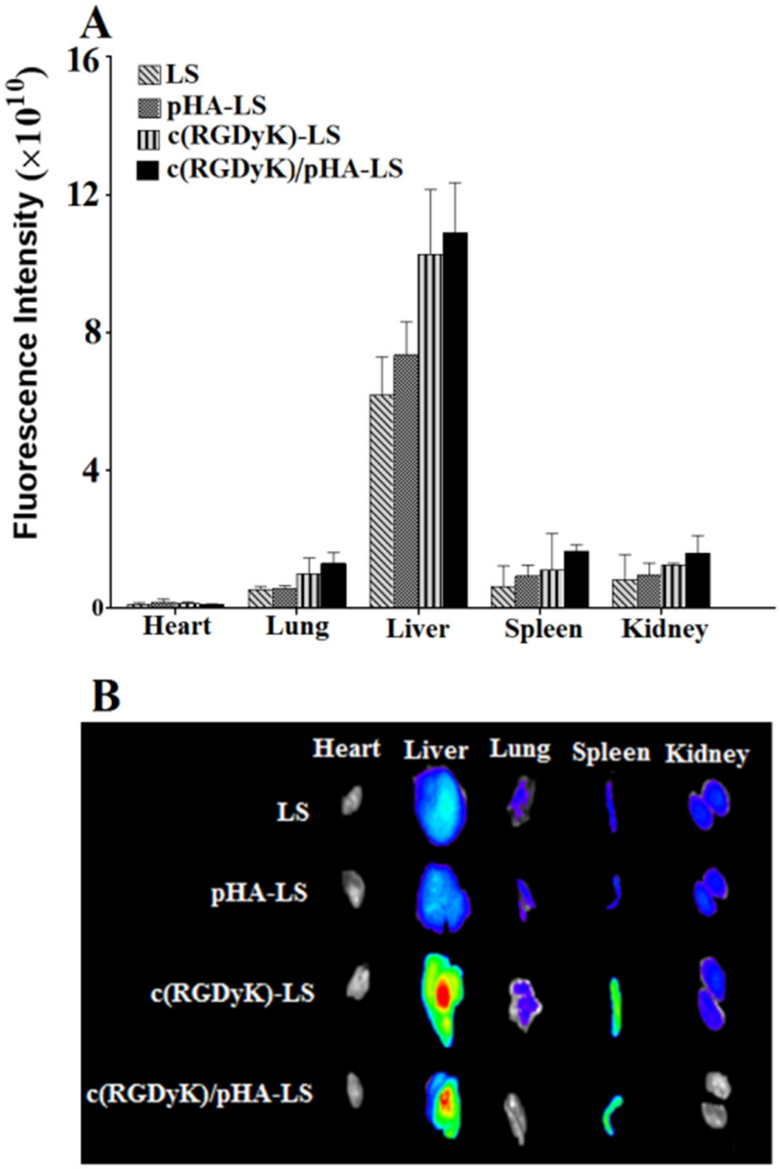

Supplementary Figure 4: *In vivo* fluorescence imaging study. Semi-quantitative analysis of the fluorescence intensity **(A)** and *ex vivo* imaging of dissected main organs 4h post injection at 15 days **(B)**. Mean ± SD, n=3.

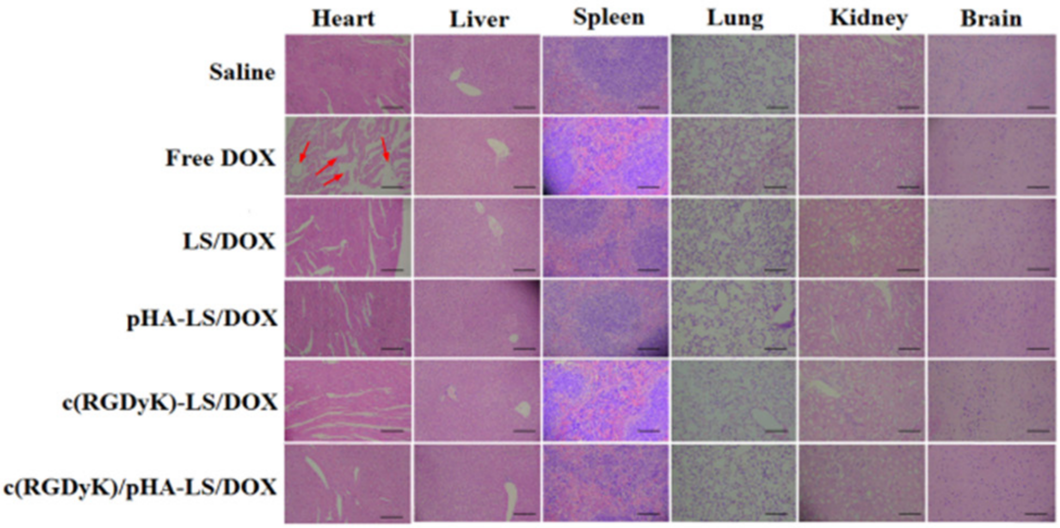

Supplementary Figure 5: *In vivo* safety evaluation. Histological analysis of the major organs of BALB/c mice after treatment with different DOX formulations. Scale bars represent 100µm.

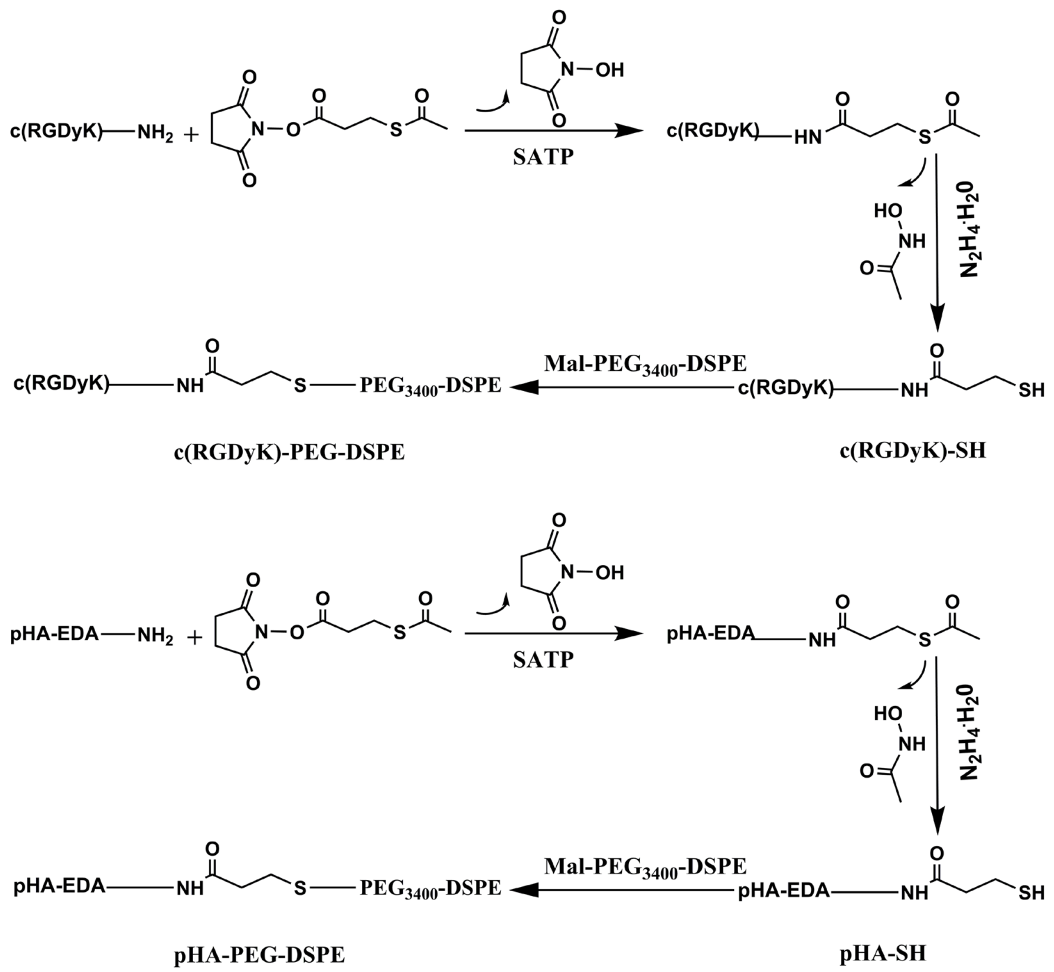

Supplementary Figure 6: Syntheses of c(RGDyK)-PEG-DSPE and pHA-PEG-DSPE. The functional materials c(RGDyK)-PEG-DSPE and pHA-PEG-DSPE were synthesized through covalent conjugation between thiolated ligands and MAL-PEG-DSPE.
